# A systematic review on reverse-zoonosis: Global impact and changes in transmission patterns

**DOI:** 10.5455/javar.2024.k810

**Published:** 2024-09-29

**Authors:** Zakaria Al Noman, Shadia Tasnim, Rony Ibne Masud, Tasnia Tabassum Anika, Md. Saiful Islam, Al Muksit Mohammad Taufiquer Rahman, Md. Tanvir Rahman

**Affiliations:** 1 Bangladesh Council of Scientific and Industrial Research, Dhaka, Bangladesh; 2 Department of Pathology, Faculty of Veterinary Science, Bangladesh Agricultural University, Mymensingh, Bangladesh; 3 Department of Microbiology and Hygiene, Faculty of Veterinary Science, Bangladesh Agricultural University, Mymensingh, Bangladesh; 4 Department of Animal Sciences, University of California – Davis, Davis, CA, USA; 5 Department of Medicine, Rajshahi Medical College, Rajshahi, Bangladesh

**Keywords:** Reverse zoonosis, zooanthroponosis, human-animal transmission, emerging infectious diseases, public health impact, globalization

## Abstract

Reverse zoonosis or zooanthroponosis is the transfer of pathogens from humans to animals. Although less studied than zoonotic diseases, this phenomenon poses significant risks to both animal and public health. The increasing human-animal interactions driven by urbanization, globalization, and environmental changes have exacerbated the occurrence of reverse zoonosis. This review evaluated the global impact and transmission patterns of reverse zoonosis, highlighting the anthropogenic and intrinsic factors contributing to its emergence. The study performed a systematic review and included 91 scientific articles published from 2000 to 2022, covering viral, bacterial, parasitic, fungal, and protozoal reverse zoonoses. This study indicated that viral infections, particularly respiratory viruses such as severe acute respiratory syndrome coronavirus-2 and influenza, have the highest incidence of reverse zoonosis, followed by bacterial infections like tuberculosis and methicillin-resistant *Staphylococcus aureus*. The United States, India, and Hong Kong are among the most reported regions for reverse zoonotic events. Major risk factors identified include environmental degradation, climate change, antimicrobial resistance, and global wildlife trade. The review underscores the need for enhanced surveillance systems, interdisciplinary collaboration, and stringent regulations on wildlife trade and animal husbandry practices to mitigate the risks associated with reverse zoonosis. Understanding the dynamics of human-animal pathogen transmission is crucial for developing not only effective but also sustainable strategies to protect animal populations as well as public health from emerging infectious diseases.

## Introduction

The term “Zoonoses” originates from the Greek words “Zoon,” meaning animal, and “nosos,” meaning disease or illness. Zoonosis is defined by the World Health Organization (WHO) as any disease or infection that can naturally be transmitted between vertebrate animals and humans, or vice versa [1]. Approximately 61% of human pathogens are of zoonotic origin [2]. The report “Asia Pacific Strategy for Emerging Diseases 2010” revealed that more than 70% of these pathogens originated from wildlife species and account for about 60% of emerging human infections [3]. Furthermore, according to recent data, there are about 1,400 human pathogens, of which 800 species are zoonotic. 130 zoonotic pathogens have been identified among the 180 pathogens that have emerged or reemerged in the past three decades [4]. Endemic zoonotic diseases disproportionately affect impoverished communities, leading to the loss of over 10 million disability-adjusted life years (DALYs) annually. In low- and middle-income countries, zoonotic pathogens are responsible for a large share of the 33 million DALYs lost to foodborne illnesses. This does not include the burden from emerging zoonotic diseases like HIV/AIDS (48 million DALYs) or coronavirus disease 2019 (COVID-19) (42 million DALYs). Overall, zoonotic diseases contribute to 25% of the 420 million DALYs lost globally to infectious diseases [5].

Globally, severe infections in humans were caused by the majority of emerging zoonotic diseases, such as highly pathogenic avian influenza, Nipah virus infection, Middle East respiratory syndrome, severe acute respiratory syndrome (SARS), COVID-19, and pandemic H1N1 (pH1N1) infection. Animals (domestic and wild) act as a reservoir for zoonotic diseases. Because of close interaction, household animals such as dairy animals, pets, and poultry transmit pathogens frequently to humans. The emergence of zoonotic diseases in humans has been attributed to anthropogenic factors, such as changes in human habitual territory and social behaviors, animal-human interaction, urbanization, modernization of landscape agriculture and animal agriculture, wild animal trade, wildlife hunting, climate change, and destruction of wild animal domain. Along with these anthropogenic factors, intrinsic elements like host-pathogen interaction with vectors also played a role in the spreading of zoonotic pathogens to people [6].

Because of its contagiousness, zoonosis has a significant economic value and has contributed significantly to the public health field, as discussed above. One often overlooked issue is reverse zoonosis, also known as anthropozoonosis, which can occasionally be confused with zooanthroponosis. This further confusion is caused by the frequent interchange between “anthropozoonosis” and “zooanthroponosis” among scientists. A 1967 Food and Agriculture Organization and WHO committee conference that advocated using the term “zoonosis” to characterize the two-way transmission of infectious pathogens between animals and humans helped clear this mistake.

Zooanthroponosis originated from the Greek words zoon, meaning “animal,” Anthropos, meaning “man”, and “nosos”, meaning “disease.” Anthroponosis is a human pathogen reservoir that can spread to non-human species [7,8]. The relationship between humans and animals is expected to grow stronger in the coming decades, driven by factors such as animal husbandry practices, rising demand for protein, the expanding companion animal market, climate change, habitat disruption, and global travel and commerce. As a result, diseases are increasingly transmitted between animals and humans. Recent research indicates that over 60% of all known infectious diseases are transmitted from animals, and around 75% of new or emerging infections in humans originate from animals [9]. It is estimated that there are around 1031 viruses on Earth, which is about 10 billion times the number of stars in the universe. While roughly 1,400 human pathogens have been identified, there are an estimated one trillion microbial species on Earth, with most remaining uncharacterized [10]. An average person carries around 30 trillion cells as well as a similar amount of bacteria, mostly located in the intestines [11].

Many infected individuals during a pandemic may act as a reserver of infection for animals experiencing zooanthroponosis. When a disease spreads from human to animal, this occurs. After the COVID-19 pandemic, this issue has gained significant attention as studies worldwide show that humans can occasionally pose a danger to animals. In addition to endangered animals, this phenomenon also impacts the human population. The population of the affected animals may develop into a virus reservoir, allowing for the reintroduction of the virus into people, which ultimately raises a concerning public health issue. Initially, the infected animals may be sick and possibly die. This issue was first highlighted during the influenza pandemic; the human pH1N1 gene was frequently found in both animals (domestic and wild) and birds, including swine, American mink, turkey, pet dogs, cats, ferrets, and even cheetahs and captive giant pandas. The scenario was fully cleared during the pandemic period of COVID-19, as human SARS the COV-2 virus frequently spreads from humans to domestic and wild animals, including a dog, cat, hamster, mink, lion, tiger, and so on, across the globe. Not only by the virus, different species of bacteria, including *Mycobacterium tuberculosis*, *Salmonella*, *Streptococcus*, and *Staphylococcus*, are frequently found to transmit from humans to animal species. According to earlier studies, there were three million fatalities and eight million new *tuberculosis* cases yearly. Solely, this illness is to blame for 6%–7% of all deaths in developing countries [12].

Despite the recent increase in studies on the adverse effects of zoonosis disease and its public health impact, limited reviews on the most prevalent 21st-century phenomenon of reverse zoonosis and its influence on animal health have been published. Therefore, this systematic review focuses on the recent increase of diseases affecting animals and wildlife by human beings and the global scene and trends. Moreover, this review analyzes the research on the recently reported cases of zooanthroponosis with an emphasis on expected worldwide patterns of its transmission.

## Materials and Methods

### Systematic review protocol

The systematic review adhered to the standard procedures outlined by the preferred reporting items for systematic reviews and meta-analyses including the relevant literature database search, evaluating their quality, data, and extractin.

### Article selection

Articles on reverse zoonosis and zooanthroponosis were gathered from PubMed and ISI Web of Science, covering research from January 2000 to December 2022. Searches used keywords such as “Zooanthroponosis,” “reverse zoonosis,” “viral reverse zoonosis,” “bacterial reverse zoonosis,” “parasitic reverse zoonosis,” and “human to animal disease transmission.” Additional articles and bibliographies were reviewed for relevant information. To capture the maximum number of relevant articles and account for variations in terminology, the asterisk (*) was used as per guidelines. After screening and removing duplicates, only studies meeting the inclusion criteria—excluding those before 2000, non-English, abstracts, and conference proceedings—were retained. The selected full articles were managed using Mendeley reference management software.

## Result and Discussion

### Details of included studies

**Figure 1.**
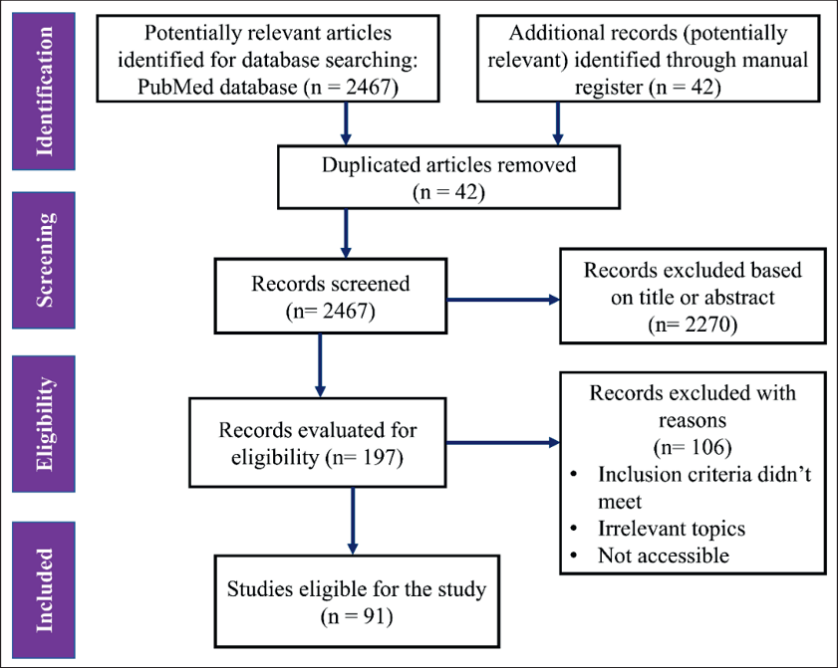
A PRISMA flow diagram depicting the study selection process. We searched PubMed online databases for studies on reverse zoonoses using predefined search algorithms. After merging the records and eliminating duplicates with Zotero software, we screened the data according to established eligibility criteria.

We initially screened 2,509 articles (2,467 from the PubMed database and 42 from other sources). After removing duplicates and excluding articles that were irrelevant or lacked sufficient detail, 197 articles were chosen for eligibility evaluation. Finally, 91 scientific articles fulfill the eligibility criteria for reverse zoonoses and were incorporated into our research project (Fig. 1).

### Reported event of reverse zoonosis

Based on reverse zoonosis, the disease can be categorized among the causal agents, including viral, bacterial, parasitic, fungal, chlamydial, and rickettsia infection. Zoonoses research focusing on fungi was being done as early as 1988. The initial study suggested that human-to-animal transmission of *Microsporum* and *Trichophyton* occurred, with a later paper focusing on *Candida albicans*. Since 1995, most research on diseases of bacterial origin that have implications for reverse zoonoses has been directed toward these infections. Since its inception in 1998, reports on reverse zoonoses involving viral infections have mainly concentrated on influenza. Finally, research has shown that human parasites can spread to animals. These studies were originally published in 2000 [13].

Even though the first reverse zoonosis was caused by a fungus, after the influenza pandemic, people could see the significance and necessity of public health. H1N1 virus, a new strain of influenza A first appeared in people in the spring of 2009, and it quickly spread around the world and the United States [14]. Concern arose when it was discovered that this human version was regularly transmitted to various animal species, including swine, turkey, domestic dogs, domestic cats, elephants, cheetahs, ferrets, pandas, and others. The severe acute respiratory syndrome Coronavirus 2 (SARS-CoV-2) virus was first detected in Wuhan, China, at the end of 2019. Since then, the outbreak has spread worldwide, with 767,972,961 confirmed cases and 6,950,655 fatalities as of 12 July 2023 [15]. Alpha B (1.1.7) was the first identification variant, spread 192 locations worldwide, and the chronologically detected Beta (B.1.351), Gamma (P.1), Delta (B.1.617.2), and Omicron (B.1.1.529) [16]. As a consequence of intimate human interaction, this variety of reverse zoonosis has spread quickly across numerous animal species worldwide. Additionally, this variety is frequently observed in the species dog, cat, tiger, lion, mink, hamster, and so on.

Table 1 presents extensive data on various instances of reverse zoonosis involving viral species with a particular emphasis on COVID-19. Numerous documented cases illustrate the transmission of COVID-19 from humans to animals in both domestic and wild species, reflecting the global scale and impact of this phenomenon. Household animals or pets, like cats and dogs, have been frequently infected through close contact with their owners in diverse regions, including the USA, Italy, Hong Kong, and Chile. These pets contracted the virus primarily through direct interactions with their infected owners, demonstrating the ease of human-to-animal transmission in domestic settings. In addition to household pets, the table highlights several instances of COVID-19 transmission to zoo animals. For example, animals of the *Falidae* family, such as tigers, lions, and snow leopards, in renowned zoological institutions like the Bronx Zoo in New York, the Louisville Zoo in Kentucky, and the Virginia Zoological Park become ill due to infection from close contact with zoo employees or visitors. These cases underscore the vulnerability of captive wildlife to human-borne diseases, emphasizing the need for stringent biosecurity measures in zoos and wildlife reserves. Farm animals, particularly mink, also experienced significant transmission events. In Spain, the Netherlands, and Denmark, numerous outbreaks of COVID-19 among mink populations were linked to close contact with farm workers and personnel. These outbreaks highlight the risks posed by intensive farming practices and the close quarters in which these animals are kept, facilitating the spread of the virus. Moreover, this includes reports of reverse zoonosis involving less commonly discussed species. For instance, wild mustelids in Brittany, France; black-tailed marmosets in Brazil; and cattle in Germany and Italy were infected with COVID-19 through direct or indirect human interactions. These cases indicate that the impact of reverse zoonosis extends beyond domestic pets and farm animals to include a wide variety of wildlife, necessitating broader surveillance and preventive measures.

Table 2 provides a comprehensive overview of bacterial species implicated in reverse zoonosis events globally during the 21st century. *Tuberculosis* is highlighted in multiple reports, including cases where *M. tuberculosis* and *Mycobacterium bovis* were transmitted from farmers to cattle and goats in South-Eastern Nigeria and central Ethiopia and from caretakers to elephants in Southern India. Additionally, human-associated *Mycobacterium orygis* was found in blackbucks and spotted deer in Chennai, India, emphasizing the varied geographical spread and species affected by this disease. Salmonellosis, caused by *Salmonella* ser. Enteritidis, was reported in sea birds and poultry in the Antarctic area, showcasing significant connectivity among human and animal populations even in remote regions. Methicillin-resistant *Staphylococcus aureus* (MRSA) was another major bacterial pathogen, with instances of transmission from animal handlers to cattle in Australia, from farm personnel to pigs and cows in Germany and the United States, and from animal handlers to livestock in Taiwan. *Escherichia coli* infections, particularly those involving extended-spectrum beta-lactamase (ESBL)-producing strains, were noted among European dogs and horses, further illustrating the breadth of reverse zoonosis. The data also include *Helicobacter pylori* infections in captive marsupials in Australia and *Campylobacter* spp. infections in human-habituated gorillas in Uganda, highlighting the diverse bacterial species and the wide array of animals impacted by human-to-animal transmission events.

Table 3 provides detailed data on reports of reverse zoonosis involving parasitic species across the globe in the 21st century. Human-associated gastrointestinal parasites such as *Strongyloides* spp., *Oesphagostomum* spp., *Trichostrongylus* spp., *Entamoeba histolytica*, and *Giardia* spp. were transmitted to mountain gorillas in Africa through direct contact. Dracunculiasis, caused by *Dracunculus medinensis*, was found to be transmitted from humans to dogs through the consumption of water fleas. Enteric parasites like *Blastocystis hominis* were detected in domestic and wild animals in Pakistan and America, indicating undefined transmission pathways. These examples illustrate the wide variety of parasitic infections that can be passed from humans to different animal species, impacting wildlife health and conservation efforts on a global scale.

Table 4 provides detailed data on various instances of reverse zoonosis involving fungal species across the globe. Microsporidiosis caused by *Encephalitozoon intestinalis* has been reported in free-ranging gorillas in Uganda, indicating the transmission of this pathogen through undefined means from humans to gorillas. Cryptosporidiosis, attributed to *Cryptosporidium hominis*, has been documented in captive flying foxes in Australia, with direct contact identified as the transmission pathway. Cases of superficial mycosis, specifically infections with *Microsporum gypseum*, have been observed in German Shepherd puppies in Jaipur, India, where the infection was linked to direct contact with itching lesions on the owner’s leg. Additionally, candidiasis caused by *Candida albicans* has been noted in parakeets in Tamil Nadu, India, where direct contact during caretaking was the mode of transmission. These instances underscore the diverse range of fungal infections that can be transmitted to various animal species from humans, highlighting the significance of direct contact in the spread of these diseases. The data reflect the global reach and varied nature of fungal reverse zoonoses, stressing the requirement for heightened awareness and preventive measures to protect both animal and human health.

**Table 1.** Reports of reverse zoonosis (zooanthroponosis) by viral species.

Disease/ descriptions	Source/Type	Infected animal	Region identified	Transmission	References
COVID-19	Pet owner	Cats and dogs	Texas, USA	Close contact	[17]
	Household	Dog, cat	Italy	Undefined	[18]
	Pet owner	Dog	Hong Kong	Close contact	[19]
	Pet owner	Cat	Santiago, Chile	Close contact	[20]
	Pet owner	Cat	Spain	Close contact	[21]
	Pet owner	Cat	Honk Kong	Close contact	[20]
	Pet owner	Cat	Belgium	Natural transmission	[22]
	Undefined	Cat	New York, USA	Undefined	[23]
	Asymptomatic SARS-CoV-2 infected humans	Stray cat	Zaragoza, Spain	Close contact	[24]
	Zoo employee	Tiger	New York, USA	Undefined	[25,26]
	Zoo employee	Tiger and lion	Bronx Zoo, New York, USA	Close contact	[27]
	Visitors or Zoo employee	Malayan tigers, Amur tigers, lions	Bronx Zoo, New York, USA	natural transmission	[28]
	Zookeeper	Snow leopards	Louisville Zoo, Kentucky, USA	Undefined	[29]
	Visitors	Free ranging leopard	India	Human contamination of environment	[30]
	Human (Delta mutant)	Asiatic lions	India	Undefined	[31]
	Handlers, Keepers, visitor	Asiatic lions	Arignar Anna Zoological Park, Chennai, India.	Undefined	[32]
	Unknown, but Probably employee or visitors	Malayan tiger	Virginia Zoological Park, USA	Undefined	[33]
	Zoo caretakers	Lions	Barcelona Zoo, Spain	Contact	[34]
	Farm workers	Mink	Spain	Direct contact	[35]
	Farm personnel	Mink	Netherlands	Direct contact	[36]
	Farm Personnel	Mink	Netherlands	Close contact	[37]
	Infected human	Mink	Denmark	Contact	[38]
	Human	White-tailed deer	USA	Undefined	[39]
	Human	White-tailed deer	Iowa, USA	Undefined	[40]
	Human (Alpha and Delta Variants)	White-tailed deer	Pennsylvania, USA	Undefined	[41]
	Human (Omicron variant)	White-tailed deer	New York, USA	Undefined	[42]
	Undefined	Pig	Tianjin, China	Undefined	[43]
	Owner	pet ferret	Slovenia	Close contact	[44]
	Zoo employee	Gorillas, Big cats	Prague Zoo, Czech- Republic	Undefined	[45]
	Zoo employee (SARS-CoV-2 delta variant)	Fishing cat Binturong, Gcoati	Chicago Zoological Society’s Brookfield Zoo, USA	Undefined	[46]
	Infected human	Wild mustelids	Brittany (France)	Indirect contact	[47]
	Unknown	Black-Tailed Marmoset	Cuiabá, Mato Grosso State, Brazil	Undefined	[48]
	Infected human	Cattle	Germany	Close contact	[49]
	Farm owner	lactating cows	Italy	Contact	[50]
	Animals under human care	Dog, Cat, Tiger, Lion, Gorilla, Leopard, Hyena, Otter, Mink	Different states, USA	Undefined	[26]
	Owner	Domestic Rabbits	France	Close contact	[51]
	Pet owner	Golden hamster	USA, Hong Kong	Direct contact	[52]
Influenza	Human	Pig	Undefined	Undefined	[54]
	pH1N1 influenza	Pig farm	Australia	Undefined	[55]
	Pig industry worker Influenza A(H1N1)	Swine	France	Contact	[56]
	Sub-clinically infected farm workers (pH1N1)	American mink	Norway	Undefined	[57]
	Farm worker	Turkey	Norway	During artificial insemination (AI)	[58]
	Pet owner (pH1N1)	Domestic dogs, cats, and pet ferrets	Sporadically	Close contact	[59–61]
	Care-takers	Cheetah	California, USA	Undefined	[62]
	Owner	Captive giant panda	Hong Kong	Close contact	[63]
	Veterinarian, Caretaker	Bornean binturong, American badger, Black-footed ferret	San Diego, California, USA	Undefined	[64]
	Mahouts, infected tourists	Asian elephants	Thailand	During elephant riding and feeding	[65]
	mink farm workers (pH1N1)	Striped skunk	Canada	Undefined	[66]
	Human (influenza H1N1)	Swine	North Vietnam	Undefined	[67]
	Human (H1N1)	Swine	South Korea	Undefined	[68]
	Human Influenza A	Wild and domestic birds, pigs, horses, bats	Undefined	Undefined	[69]
	human HEV (strain TW6196E)	Pig	USA	Experimental	[70]
Rota virus	Human RAC-DG5 and MP-CIVET66 strains	Japanese raccoon dog, masked palm civet	Japan	Undefined	[712010]
Mumps virus	Human paramyxovirus	Dog	Undefined	Undefined	[72]
Arbovirus	Human (Yellow fever, Dengue, Chikungunya and Zika viruses)	Animal	Kenya	Bite by *Aedes aegypti*	[73]
Chikungunya virus	Human and other primates	Non-human primates	Sporadically	Bite by *Aedes albopictus*	[74]
Dengue virus	Human	Wild mammals	French Guiana	Aedes spp. bite	[75]
Human adenovirus	Human	Non-human primate, bat, feline, swine, canine, ovine, and caprine	USA, Gabon, China, Tanzania, Thailand, Uganda, Rwanda	Undefined	[76]
Meta-pneumo virus	Human population	chimpanzees	Mahale Mountains National Park, Western Tanzania	Undefined	[77]

**Table 2.** Reports of reverse zoonosis (zooanthroponosis) by different bacterial species across the globe in the 21st century.

Disease/ descriptions	Infection Source	Species	Infected animal	Region identified	Transmission	References
Tuberculosis	Farmer	*M. tuberculosis, M. bovis*	Cattle, Goat	South-Eastern Nigeria	Human-animal contact	[78]
	Farmer	*M. tuberculosis M. bovis M. africanum*	Cattle	Nigeria	Cohabitation	[79]
	Farmer	*M. bovis*	Cattle	Poland	Direct contact	[80]
	Farmer	*M. tuberculosis*	Cattle	Central Ethiopia	Close Contact	[81]
	Caretaker	*M. tuberculosis*	Elephants	Southern India	Close Contact	[82]
	Animal caretaker	*M. tuberculosis*	Dairy Cattle	North India	Close Contact	[83]
	Human	*M. orygis*	Blackbucks, Spotted deer	Chennai, India	Human-animal interaction	[84]
Salmonellosis	Human	*Salmonella *ser*. *Enteritidis	Sea bird, poultry	Antarctic area	Substantial connectivity among populations	[85]
	Pet owner	*Salmonella *spp*.*	Dog	Kosice, Slovakia	Feed contamination	[86]
Staphylococcal infections	Animal handler	MRSA	Cattle	Australia	Direct Contact	[87]
	Farm personnel	MRSA	Cattle, Pig	German	Direct Contact	[88]
	Human	MRSA	Cows, turkeys, pigs	United States of America	densely concentrated animals	[89]
	Animal handler	Oxacillin-resistant *Staphylococcus aureus* (ORSA)	Livestock	Taiwan	Contact	[90]
*E. coli* infections	Animal owner	ESBL-producing *E. coli*	Dog, Horse	European-wide service area	Direct Contact	[91]
*Helicobacter* infections	Animal Handler	*Helicobacter pylori*	Captive marsupial, Dunnart	Australia	Direct Contact	[92]
Campylobacteriosis	Human	*Campylobacter *spp.	Human habituated gorilla	Uganda	Unknown	[93]
*Pseudomoniasis*	Pet owner	*Pseudomonas aeruginosa*	Pet dog	Brazil	Direct contact, hospital discharge	[94]

Table 5 provides a comprehensive overview of protozoal species implicated in reverse zoonosis events across the globe. The table highlights several significant instances where protozoal infections have been transmitted from humans to various animal species. *Leishmania* species, specifically *L. donovani* and *L. braziliensis*, have been reported in dogs, foxes, and rodents in regions such as South Sudan, India, and Bangladesh through close human contact. Giardiasis, caused by *Giardia duodenalis*, has affected stray, semi-stray, and domestic cats in Iran, where the transmission was linked to close contact with pet owners. In Australia, *G. duodenalis* has also been found in African-painted dogs due to close interaction and runoff of human sewage upstream. Furthermore, in Fiema, Ghana, colobus monkeys were infected with both *G. duodenalis* and *Isospora belli*, although the exact transmission pathways were undefined. Notably, malaria caused by *Plasmodium simium* has been identified in platyrrhine monkeys in America, with mosquitoes acting as vectors for the disease transmission. These cases illustrate the diverse range of protozoal infections that can be spread from human to animal, stressing the requirement of better awareness and preventive strategies to protect animal health from these parasitic diseases.

**Table 3.** Reports of reverse zoonosis (zooanthroponosis) by different parasitic species across the globe in the 21st century.

Disease/ descriptions	Source	Species	Infected animal	Region identified	Transmission	References
Gastrointestinal parasite infection	Human	*Strongyloides* spp., *Oesphagostomum* spp., *Trichostrongylus* spp., *Entamoeba histolytica, Giardia* spp.	Mountain gorillas	Parc National des Volcans, Rwanda	Direct contact	[95]
Dracunculiasis	Human	*Dracunculus medinensis*	Dog		Water fleas, food	[96]
*Enteric parasite infection*	Human	*Blastocystis hominis*	Livestock and wild animals	Pakistan, America	Undefined	[97]

**Table 4.** Reports of reverse zoonosis (zooanthroponosis) by different fungal species across the globe in the 21st century.

Disease/ descriptions	Source	Species	Infected animal	Region identified	Transmission	References
Microsporidiosis	Human	*Encephalitozoon intestinalis*	Free range gorilla	Uganda	undefined	[98]
Cryptosporidiosis	Human	*Cryptosporidium hominis*	Captive Flying fox	Australia	Direct contact	[99]
Superficial mycosis	Owner	*Microsporum gypseum*	German shepherd puppy	Jaipur, India	Direct contact with itching lesions on the owner‘s leg	[100]
Candidiasis (Fungal)	Pet owner	*Candida albicans*	Parakeet	Tamilnadu, India	Direct contact during caretaking	[101]

**Table 5.** Reports of reverse zoonosis (zooanthroponosis) by different protozoal species across the globe in the 21st century.

Disease/ descriptions	Source	Species	Infected animal	Region identified	Transmission	References
Leishmaniasis (Protozoal)	Human	*L. donovani L. braziliensis*	Dog, fox, rodent	South Sudan, India, Bangladesh	Human close contact	[102,103]
Giardiasis (Protozoal)	Pet owner	*Giardia duodenalis*	stray, semi-stray, domestic cats	Iran	Human close contact	[104]
	Zookeepers	*Giardia duodenalis*	african painted dog	Australia	Close interaction, Runoff of human sewage upstream	[105]
Isosporiosis and Giardiasis (Protozoal)	Human	*Giardia duodenalis *and* I. belli*	colobus monkeys	Fiema, Ghana	Undefined	[106]
Malaria (Protozoal)	Human	*Plasmodium simium*	Platyrrhine monkeys	America	Mosquitoes	[107]

### Country-wise case report

“Zooanthroponosis spillover” refers to transmitting infectious agents (such as pathogens) from humans to animals, resulting in disease outbreaks among animal populations. This concept is somewhat similar to zoonotic spillover, where pathogens move from animals to humans (e.g., zoonotic diseases like COVID-19, Ebola, and so on), but the transmission direction is reversed in this case.

Zooanthroponosis, or transmission of diseases from human to animal, can have a significant effect on both animal health and conservation efforts. However, this area of research is less studied than zoonotic diseases. In spillover report, USA has been ranked top (21; 23.07%), followed by India (9; 9.89%), Hongkong (5; 5.49%), Australia (4; 4.39%), Brazil (3; 3.29%), France (3; 3.29%), Germany (3; 3.29%), Spain (3; 3.29%), and Uganda (3; 3.29%), where Canada, China, Italy, the Netherlands, Nigeria, Norway, Rwanda, and Thailand have been reported two papers (2.29%), as well as other countries, revealed at least one (1.09%) paper.

Human activities, behaviors, and interactions with animals can lead to the transmission of diseases to animal populations. Transmission could include animals living close to human settlements, interaction with domesticated animals, or exposure to human waste. The socio-economic cultures of different continents play a vital role in zooanthroponosis spillover. Most outbreaks have been reported in Asia (27.78%), followed by North America (25.56%), Europe (22.22%), Africa (14.44%), South America (5.56%), and Australia (4.44%).

### Organism-wise case report

**Figure 2.**
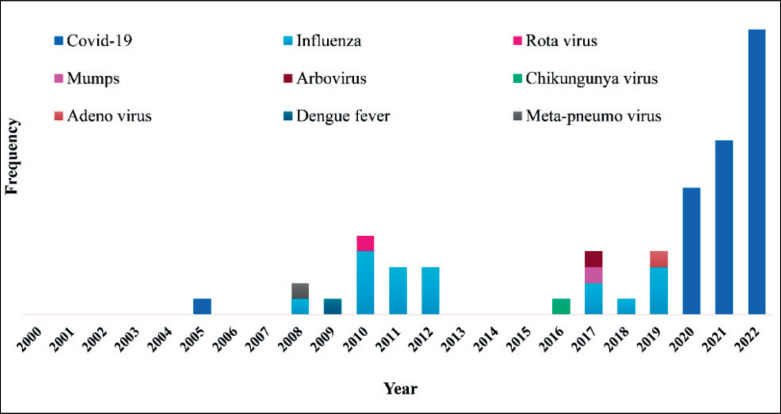
Viral reverse zoonosis outbreaks in the 21st century.

Viral pathogens are the most important infections due to their global impact, the potential for rapid spread, the diversity of diseases, challenges in treatment and prevention, and their role in shaping public health, economies, and scientific research. A virus is a type of microorganism that can cause a wide range of diseases in various living organisms such as humans, animals, plants, and even bacteria. A competitive outbreak for reverse zoonosis has been visualized in Figure 2, where respiratory viruses (COVID-19, Influenza, and so on) ranked top.

**Figure 3.**
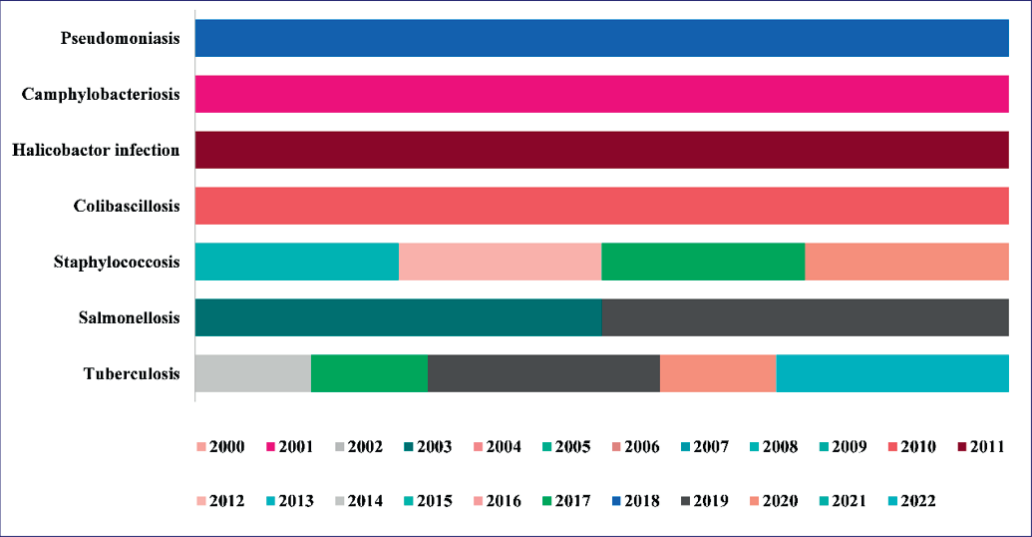
Timeline (2000–22) of bacterial outbreaks recorded as zooanthroponosis.

Bacterial infections are an integrated part of life due to the complex and intertwined relationship between bacteria and living organisms, including humans. Most bacterial interactions are harmless or beneficial; some can lead to infections and illnesses. While bacterial infections can lead to diseases, they also play vital roles in maintaining life balance and contribute to the diversity and complexity of living systems. Understanding bacterial infections, their mechanisms, and methods for prevention and treatment is crucial for maintaining public health and addressing the challenges posed by bacterial pathogens. The bacterial outbreaks are shown in Figure 3.

Parasitic infections are also important pathogens due to their ecological, evolutionary, and biological significance. While some parasites can cause diseases, others are essential components of ecosystems, contributing to biodiversity, nutrient cycling, and the intricate relationships that exist between organisms in nature.

Fungi can cause a variety of infectious diseases, impact human health, have a significant impact on agricultural and ecological systems, and have the potential to develop drug resistance.

### Timeline of reported cases

**Figure 4.**
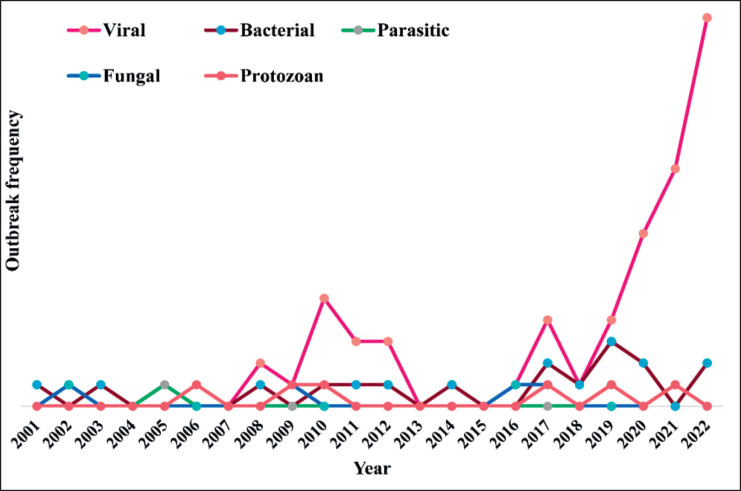
Skewness trending of reverse-zoonosis reported around the world in terms of 2000–22.

The severity of outbreaks depends on various factors such as pathogen type, geography, healthcare infrastructure, and population susceptibility. Efforts to combat these pathogens involve a combination of prevention, early detection, effective treatment, and ongoing research to understand their biology and modes of transmission better. In the spillover analysis, viral outbreaks have been trending upward, followed by bacterial, fungal, and protozoan outbreaks, whereas sporadic peaks of parasitic infections have been visualized in Figure 4.

### Major risk factors

Due to globalization, reverse zoonosis, the transmission of diseases from humans to animals, has become increasingly recognized [108,109]. Globalization is a multifaceted process transforming the world into a closely linked global village. It involves the exchange of goods, services, knowledge, and culture across international boundaries, facilitated by advancements in transportation, communication, and technology. It has led to the expansion of multinational corporations, the rise of global supply chains, and the proliferation of cross-border interactions in various domains, including economics, politics, culture, and health. The intensification of international trade in wildlife for consumption, traditional medicine, and the exotic pet trade has further heightened the risk of reverse zoonotic transmission [110–112]. Additionally, human encroachment into natural habitats, driven by factors such as urban expansion and agricultural development, brings humans closer to wildlife, thereby increasing the chance of disease emission in both directions [6, 113–115].

Urbanization, deforestation, agricultural expansion, and pollution are the major driving environmental factors for reverse zoonosis. These activities often bring humans into closer contact with wildlife, increasing the risk of disease transmission [116,117]. The encroachment of human settlements into forested areas has been linked to outbreaks of diseases such as the Ebola virus in great apes and bats [118–120]. Pollutants can degrade ecosystems and compromise the health of wildlife, making them more susceptible to infections. Moreover, certain chemicals can weaken immune systems, making animals more vulnerable to pathogens transmitted from humans [121,122].

The drug-resistance phenomenon is also accelerating reverse zoonosis. The excessive use, misuse, and overuse of antibiotics in human healthcare contribute to the rise and dissemination of drug-resistant bacteria, which can then be passed to animals through direct contact or environmental exposure. This transfer of drug-resistant pathogens to animals poses challenges for veterinary treatment and public health, as it limits the effectiveness of antimicrobial therapies in animals and may serve as a harbor for resistant bacteria [123–125].

Trade and travel exacerbate the spreading of pathogens into animal populations, posing significant threats to wildlife and domestic species [126]. One notable example is the transmission of influenza viruses from humans to various animal species, including pigs and birds [108,127]. The global trade in live animals and animal products provides ample opportunities for these viruses to spread [116,128,129].

Research suggests that climate change can exacerbate reverse zoonosis through various mechanisms, such as habitat alteration, animal behavior, and distribution changes, human-animal interactions, disease-transmitting patterns, and so on. Changes in temperature and rainfall may influence the prevalence and distribution of vector-borne diseases like malaria and dengue fever, which can spill over from humans to other animals [130–133].

Human-associated pathogens such as influenza viruses, *tuberculosis* bacteria, and antibiotic-resistant bacteria have been identified in various animal species, posing risks to wildlife and domestic animals [134–136]. The transmission of pathogens from humans to animals can lead to the establishment of novel infections in animal populations, with the potential to cause outbreaks and impact wildlife conservation efforts [137,138]. An important instance is the spread of the SARS-CoV-2 virus from people to captive and wild animals, such as large felines in zoos and mink on fur farms [27,37,139].

Reverse zoonosis is a complex phenomenon with implications for both animal and human health. One crucial aspect of this phenomenon is its correlation with the host’s nutrition status. Studies have highlighted the intricate interplay between malnutrition and the transmission of diseases from humans to animals. Malnourished individuals are more likely to shed pathogens, thereby increasing the risk of reverse zoonotic transmission to animals [123,124,140]. Addressing nutritional deficiencies in both human and animal populations is crucial for mitigating the risks associated with reverse zoonosis and promoting overall health and well-being.

Pathogen variability and mutationality also complicate the dynamics of reverse zoonosis. Pathogens such as SARS-CoV-2 viruses exhibit high levels of variability due to their rapid mutation rates, allowing them to adapt to new hosts and environments [141,142]. This mutagenic potential poses challenges for predicting and controlling the spread of diseases across species boundaries [143,144].

Vector-borne diseases also play a significant role in reverse zoonosis. Vectors such as mosquitoes, ticks, and fleas can transmit pathogens between humans and animals, leading to the establishment of new transmission cycles [37,145,146]. Lyme disease, caused by the bacterium *Borrelia burgdorferi*, can be transmitted between humans and animals via the bite of infected ticks [147]. Likewise, the West Nile virus, spread by mosquitoes, can infect various vertebrate hosts, such as humans, birds, and horses [148,149].

Several factors contribute to this reverse zoonosis phenomenon, including globalization, environmental pollution, climate change, drug resistance, host-pathogen interface, vector variation, and organism mutagenicity. These include human behavior, cultural practices, population density, healthcare-associated infections, waste management, and others.

## Conclusion

A comprehensive approach is necessary to improve animal welfare and minimize reverse zoonosis risk. Enhancing the surveillance system to monitor disease transmission between humans and animals is essential, as it involves early detection, rapid response, and effective communication between human and veterinary health sectors. Implementing and enforcing regulations that govern practices such as wildlife trade, animal husbandry, and food production can help reduce the opportunities for spreading pathogens from humans to animals. Furthermore, it is essential to advocate for the responsible use of antibiotics in both human healthcare and agriculture to address the growing issue of antibiotic resistance, which is a major risk to the health of humans and animals. Investing in public health infrastructure, including sanitation, hygiene education, and access to healthcare for both humans and animals, can help prevent the transmission of diseases in communities. Addressing socio-economic disparities and promoting sustainable development can alleviate some underlying factors driving reverse zoonosis, such as habitat destruction and urbanization. Ultimately, it is crucial to promote collaboration across different fields such as science, policy-making, healthcare, and community engagement to create comprehensive approaches to lower the chances of reverse zoonosis and safeguard both human and animal communities against infectious illnesses.

## List of Abbreviations

COVID-19, Coronavirus Disease 2019;

DALY, Disability-adjusted life year; ESBL, Extended-spectrum beta-lactamase; MRSA, Methicillin-Resistant *Staphylococcus Aureus*; pH1N1, Pandemic H1N1 Influenza Virus; PRISMA, Preferred reporting items for systematic reviews and meta-analyses; SARS, Severe acute respiratory syndrome; SARS-CoV-2, Severe acute respiratory syndrome Coronavirus 2; WHO, World Health Organization.
